# Sanger Validation of High-Throughput Sequencing in Genetic Diagnosis: Still the Best Practice?

**DOI:** 10.3389/fgene.2020.592588

**Published:** 2020-12-02

**Authors:** Rosina De Cario, Ada Kura, Samuele Suraci, Alberto Magi, Andrea Volta, Rossella Marcucci, Anna Maria Gori, Guglielmina Pepe, Betti Giusti, Elena Sticchi

**Affiliations:** ^1^Department of Experimental and Clinical Medicine, University of Florence, Florence, Italy; ^2^Department of Information Engineering, University of Florence, Florence, Italy; ^3^Atherothrombotic Diseases Center, Careggi University Hospital, Florence, Italy

**Keywords:** high-throughput sequencing, next generation sequencing, Sanger sequencing, sequencing validation, discrepancy, allelic drop-out, variant call quality

## Abstract

Next-generation sequencing (NGS)’s crucial role in supporting genetic diagnosis and personalized medicine leads to the definition of Guidelines for Diagnostic NGS by the European Society of Human Genetics. Factors of different nature producing false-positive/negative NGS data together with the paucity of internationally accepted guidelines providing specified NGS quality metrics to be followed for diagnostics purpose made the Sanger validation of NGS variants still mandatory. We reported the analysis of three cases of discrepancy between NGS and Sanger sequencing in a cohort of 218 patients. NGS was performed by Illumina MiSeq^®^ and Haloplex/SureSelect protocols targeting 97 or 57 or 10 gene panels usually applied for diagnostics. Variants called following guidelines suggested by the Broad Institute and identified according to MAF <0.01 and allele balance >0.2 were Sanger validated. Three out of 945 validated variants showed a discrepancy between NGS and Sanger. In all three cases, a deep evaluation of the discrepant gene variant results and methodological approach allowed to confirm the NGS datum. Allelic dropout (ADO) occurrence during polymerase chain or sequencing reaction was observed, mainly related to incorrect variant zygosity. Our study extends literature data in which almost 100% “high quality” NGS variants are confirmed by Sanger; moreover, it demonstrates that in case of discrepancy between a high-quality NGS variant and Sanger validation, NGS call should not be *a priori* assumed to represent the source of the error. Actually, difficulties (i.e., ADO, unpredictable presence of private variants on primer-binding regions) of the so-called gold standard direct sequencing should be considered especially in light of the constantly implemented and accurate high-throughput technologies. Our data along with literature raise a discussion on the opportunity to establish a standardized quality threshold by International Guidelines for clinical NGS in order to limit Sanger confirmation to borderline conditions of variant quality parameters and verification of correct gene variant call/patient coupling on a different blood sample aliquot.

## Introduction

Since their first appearance in the mid-2000s, next-generation sequencing (NGS) technologies have marked a new era in investigating genetic complexity. Their continuous implementation is universally perceived as crucial for their increasingly important contribution in genetic research as well as in the diagnostic field and for the overall benefit they can represent for patients management (Goodwin et al., 2016). Widely used NGS platforms have in common the ability to perform and capture data deriving from a *massively parallel sequencing* of millions of reactions simultaneously (Mardis, 2008). Continuous and impressive developments in these technologies are offering the opportunity to interrogate changes in DNA and RNA molecules at such a level of precision that the role of NGS in supporting the clinical management of patients and in personalized medicine has been addressed, thus leading to the *Guidelines for Diagnostic Next Generation Sequencing* (Matthijs et al., 2016) by the European Society of Human Genetics (ESHG). One of the main statements of the document focuses on the challenges these technologies bring in terms of data management and on acceptable, standardized validation of the tests, as NGS is not an error-free technique. Apart from those factors regarding the starting material (e.g., quality and storage conditions of the starting biological material as well as molecules interfering with DNA extraction from blood of patients treated with drug or medication) or the analytical confounders [GC- and AT-rich areas, repeat sequences, large (>20-bp) deletions (Ilyas, 2017)], a significant issue in NGS is essentially represented by the accuracy of the different bioinformatic pipelines in (a) filtering low-quality reads, (b) discriminating clinically relevant variation from background “noise” (due, for instance, to spontaneous PCR errors and deamination), and (c) accurately aligning the reads to a reference sequence. Also, a low read depth (below 10 reads per base on average for whole-exome sequencing on Illumina platforms, 20–30 reads for gene panel sequencing on Ion Torrent and Illumina) determines false-positive results due to sequencing errors especially in GC-rich regions (McCourt et al., 2013; Strom et al., 2014; Beck et al., 2016; Zheng et al., 2019). Further, clinically relevant mutations can be missed (false-negative results) as some exons may be not completely represented or sufficiently covered (Sikkema-Raddatz et al., 2013; Beck et al., 2016). For those reasons, together with the paucity of internationally accepted regulatory guidelines providing specified NGS quality metrics to be followed in the clinical setting, Sanger sequencing validation (costly, time consuming, and not error-free) of NGS detected variants is mandatory in routine diagnostics [ESGH, Matthijs et al., 2016; NGS sequencing, indications for clinical application, Società Italiana di Genetica Umana (SIGU), 2016]. However, the necessity of validating genetic variants identified through high-performance instruments, which are increasingly accurate and affordable, with a more expensive technology, raised a question about the actual cost-effectiveness of such an approach, both in research and in diagnostic fields. Several recent reports suggested that NGS data, reaching established quality scores, are as accurate as Sanger sequencing (McCourt et al., 2013; Sikkema-Raddatz et al., 2013; Strom et al., 2014; Baudhuin et al., 2015; Beck et al., 2016; Zheng et al., 2019) as the latter was able to confirm nearly 100% of NGS detected variants at an initial or a second sequencing run. A low number of NGS variants were not validated even after a second Sanger run. These variants were found to show relatively low-quality scores for their NGS calls. In the current study, we analyzed three cases of discrepancy between the NGS-generated datum and the subsequent first round of Sanger validation. Discrepant data resulted from the validation of 945 rare genetic variants identified in 218 patients.

## Materials and Methods

### DNA Isolation

Genetic analysis was conducted on a cohort of 218 patients admitted to the Advanced Molecular Genetics Laboratory, Department of Experimental and Clinical Medicine, Careggi Hospital-University of Florence. Informed consent was obtained from subjects included in the study, and the study was approved by the Careggi Hospital Ethics committee (registration number CEAVC OSS 16.291 and 11114).

Peripheral venous blood was collected in EDTA-coated vacutainer tubes and was stored at −20°C. Genomic DNA was extracted from blood samples using the Tecan^®^ Freedom EVO^®^ liquid handling platform (Tecan Group Ltd., Switzerland) and GeneCatcher^TM^ gDNA 0.3–1 mL Blood Kit (Thermo Fisher Scientific, United States). All procedures were performed following the manufacturer’s protocols.

### Next-Generation Sequencing and Variant Selection

A targeted NGS was performed on three genes panels [97 (627,782 kbp) + 57 (241,143 kbp) + 10 (49,312 kbp) genes, Supplementary Tables 1–3] applied in the diagnostic and research routine for Marfan syndrome and related disorders, familial dyslipidemia, and von Willebrand disease at the Department of Experimental and Clinical Medicine, Section of Critical Medical Care and Medical Specialities, University of Florence, Italy. Oligo probes specific for the target gene regions were designed using Agilent Sure Design (Agilent Technologies, Santa Clara, CA, United States) in order to create a custom target enrichment library of the selected genes. The capture region comprised all coding exons and flanking intron sequences (50 bp upstream and downstream at exon–intron junctions). Amplicon sequencing libraries were prepared from 50 to 750 ng of DNA per sample according to the SureSelect^*QXT*^ (for largest panel) and HaloPlexHS (for 57 and 10 gene panels) Amplicon protocol (Illumina Inc., San Diego, CA, United States). The pooled libraries were paired-end and sequenced on a micro flow cell with V3 chemistry on a MiSeq instrument (Illumina Inc., San Diego, CA, United States). The analytical pipeline available in our laboratory was developed, implemented, and validated for data analysis of targeted sequencing for diagnostic purposes. Fastq files’ quality was checked with FASTQC. Adapters and quality trimming were performed using Surecall Trimmer. Trimmed reads were aligned to the human reference genome (Human GRCh37/hg19) using BWA-MEM. Bam files’ quality was evaluated with Qualimap. Variant calling was performed using GATK4 HaplotypeCaller in GVCF mode and the joint genotyping tool GenotypeGVCFs. Variants were annotated using VEP 99. Ninety-nine percent of targeted regions were covered. Variants were filtered according to the phred quality score (Q) ≥30 and a minimum coverage depth of 30×. Variants called following guidelines suggested by the Broad Institute, commonly accepted as standard, and identified according to (a) MAF <0.01; (b) the potential pathogenetic/modulatory role, according to variant classification recommendation (Richards et al., 2015), literature genotype–phenotype association data and/or biological plausibility; (c) *in silico* predictor tools (SIFT^1^; PROVEAN^2^; PolyPhen-2^3^; MutationTaster^4^; FATHMM^5^; Human Splicing Finder^6^; NetGene2^7^); (d) type of genetic variants; (e) localization (exonic, splicing regions variants); and (f) allele balance >0.2 were Sanger validated.

### Validation by Sanger Sequencing

Specific flanking intronic primer pairs for the selected NGS variants to be validated were designed using the Primer3 algorithm^8^, one of the most widely used primer designing tools (Kumar and Chordia, 2015; Supplementary Table 4). Primer sequences were checked for the presence of single-nucleotide polymorphism (SNPs) on their complementary DNA strands (visual inspection of sequences/SNP database)^9^. The resulting amplicons were checked for sequence similarity throughout the human genome using the Primer-BLAST tool^10^. The PCR amplicons were then purified and Sanger sequenced. PCR was performed in a final volume of 25 μl using FastStart^TM^ Taq DNA Polymerase, 5 U/μl Kit (Roche), 1 μl of dNTPs (2.5 mM), 0.5 μl of each primer with a concentration of 10 pmol/μl (Eurofins Scientific, Luxembourg), and 2 μl genomic DNA in a concentration of approximately 50 ng/μl. Amplicons were purified using an Exonuclease I 20 U/μl/FastAP Thermosensitive Alkaline Phosphatase 1 U/μl (Thermo Fisher Scientific, United States) mixture. Purified PCR products were sequenced using the BigDye Terminator Kit v1.1 (Thermo Fisher Scientific, United States) and ABI 3500Dx Sequencer (Applied Biosystems, Foster City, CA, United States).

## Results

Among the total of 945 variants identified by NGS and selected for Sanger validation, 942 (99,7%) were confirmed. The mean coverage of experimental sessions was 173× for the SureSelect approach and 1100× for the Haloplex approach with an at least 98% of analyzable target bases; all variants met the phred-scaled quality score Q ≥30. Three out of 945 variants (0.3%) showed a discrepancy between the NGS datum and the subsequent validation. Two variants were in the *LTBP2* gene while the third one involved the *TGFB1* gene. All variants’ discrepancies were related to their heterozygous/homozygous state. General characteristics of mutations are reported in Table 1. The depth of coverage for the three loci ranged from 173× to 199× (Table 1). All 3 variants were called as heterozygous and presented with balanced reads containing the wild-type or mutant allele (percentages of mutant on total alleles range from 45 to 54%).

**TABLE 1:** Discrepant genetic variants analyzed in the study.

	**Gene**	**Variant description**	**dbSNP**	**MAF_EU (ExAC)**	**Chromosomic position (reads number per allele)**	QUAL*
P1	*LTBP2* NM_000428	c.3979C > T p.Arg1327Cys	rs758023418	0.0000088	chr14_74973455_C/T (0/1:107,92)	3197.33
P2	*LTBP2* NM_000428	c.4203G > A p.Thr1401=	rs150977380	0.00428	chr14_74971852_C/T (0/1:96,103)	4613.20
P2	*TGFB1* NM_000660	c.169C > A p.Leu57Met	rs1203938760	0.0000649	chr19_41858781_G/T (0/1:96,77)	2060.33

*phred-scaled quality score. MAF, minor allele frequency; EU, European.

**FIGURE 1:**
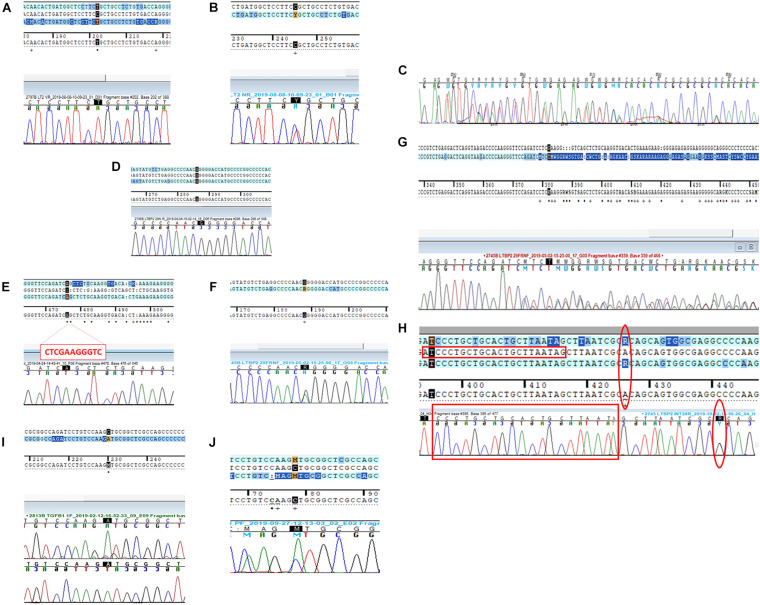
Direct sequencing electropherograms of the genetic variants in **(A)** *LTBP2* (P1), original primers; **(B)** *LTBP2* (P1), new primers; **(C)** *LTBP2* deletion (P1), new primers; **(D)** *LTBP2* point mutation (P2), original primers; **(E)** *LTBP2* deletion (P2), original primers; **(F)** *LTBP2* point mutation (P2), second F primer + original R primer; **(G)** *LTBP2* deletion (P2), second F primer + original R primer; **(H)** *LTBP2* (P2) intronic primers (the empty red rectangles indicate the original F primer sequence; the empty red circles indicate the rs11846588 SNP proximal to the 3′ primer end); **(I)** *TGFB1* (P3), original primers; and **(J)** *TGFB1* (P3), new primers.

*Patient 1 (P1)* The first variant showing a discrepancy between the NGS call and the subsequent Sanger validation involved the *LTBP2* gene (latent transforming growth factor-beta-binding protein 2, OMIM^∗^602091). The missense *LTBP2* mutation, NM_000428.3:c.3979C > T (p.Arg1327Cys, rs758023418), had a MAF of 0.0000088 in the ExAC database for the European population. Primers were designed as previously described (Supplementary Table 4). The Sanger electropherogram in Figure 1A only revealed the presence of the mutated base (T at 3979 locus). The primer-binding regions were therefore checked for the presence of SNPs on their complementary DNA sequences and found negative. A second primer pair, external to the first ones, was designed and used for the direct sequencing of the new amplicons. The resulting electropherograms (Figure 1B) showed the point mutation in the heterozygous state (as called by NGS) as well as a 2-bp deletion within the forward primer-binding DNA region (NM_000428.3:c.3908-98_3908-97delAG, rs149267227, Figure 1C). This intronic variant had a MAF in the European population of 0.0029.

**FIGURE 2:**
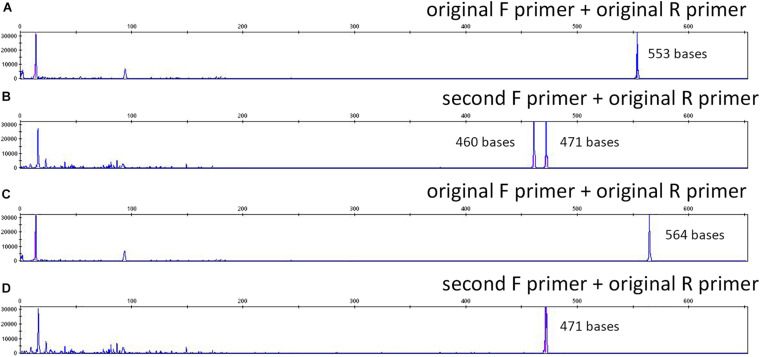
Capillary electrophoresis results using 6-FAM-labeled original and external forward primers for *LTBP2* variant amplification (P2): **(A)** P2, original F primer; **(B)** P2, second F primer **(C)** negative control, original F primer; **(D)** negative control, second F primer. Peaks sizes are reported (red).

*Patient 2 (P2) LTBP2* second variant (NM_000428.3:c.4203G > A) was a synonymous mutation (p.Thr1401=) with a MAF of 0.00428 in the European ExAC population. Primers were constructed, and the electropherogram, deriving from the direct sequencing, did not reveal the point mutation (only the wild-type base was present) (Figure 1D); in addition, an 11-bp deletion was identified at the homozygous state (Figure 1E). The combination of a second forward primer and the original reverse one generated amplicons whose electropherograms revealed both the mutation and the deletion in the heterozygous state, as they were called by NGS (Figures 1F,G; a schematic representation of the PCR results and the primer localization on the DNA sequence is reported in Supplementary Figure 1). Afterward, in order to provide a further confirmation of data obtained, we performed capillary electrophoresis using 6FAM labeled oligonucleotides for both the original and the second forward primers. Amplicons derived from the two different primer combinations (original F primer + original R primer vs. second F primer + original R primer) were analyzed along with a negative control (a DNA sample belonging to the same experimental session and in which the NGS call of the 11-bp deletion was negative). As shown in Figure 2A, the original primer pair generated a single peak, whose size matched the allele containing the 11-bp deletion, which underwent a preferential amplification. On the other hand, two different peaks resulted from the combination of the second F primer + the original R one, whose sizes, 460 and 471 bp, were consistent with the presence of the 11-bp deletion in that amplicon in the heterozygous state (Figure 2B). As concerns the negative control, resulting peaks confirmed the absence of the 11-bp deletion (Figures 2C,D). These data were consistent with Sanger electropherograms deriving from the combination of the second F primer and the original R one, in which the 11-bp deletion was detected in the heterozygous state (Figure 1G). Ultimately, a third, intronic, primer pair was designed, in order to analyze the sequence of the original forward primer. The resulting electropherogram was negative although it actually revealed the presence of an SNP [NM_000428.3:c.4178-224A > G, rs11846588, MAF_EU(ExAC) = 0.16] proximal to the 3′ primer end which was not previously detected (Figure 1H).

**FIGURE 3:**
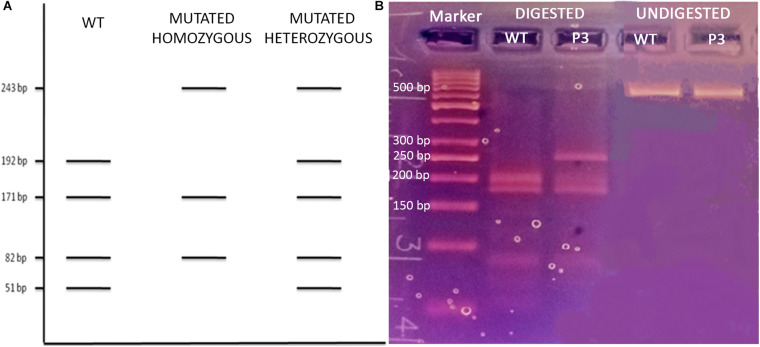
**(A)** Schematic representation of the AluI enzyme digestion on a wild-type, homozygous, and heterozygous mutated *TGFB1* fragment; **(B)** 3% agarose gel electrophoresis of the AluI enzyme digested and undigested P3 and WT control.

*Patient 3 (P3)* The last analyzed variant was a missense mutation (NM_000660.6:c.169C > A, p.Leu57Met, rs1203938760) involving the *TGFB1* gene (transforming growth factor-beta-1, OMIM^∗^190180) with a MAF in the European ExAC population of 0.0000649. We proceeded with the Sanger validation ultimately obtaining an electropherogram showing the variant in the homozygous state (Figure 1I). Once again, we tested the primer pair used for the initial amplification for their capacity to bind to SNPs and they resulted negative. We therefore checked the experimental steps (initial amplification or direct sequencing) in order to unequivocally identify the phase in which the allelic loss took place. A restriction fragment length polymorphism (RFLP) procedure was performed using AluI enzyme cutting at a specific cleavage DNA site (5′…AG ↓CT…3′), which was identified by the NEBcutter V2.0^11^ online tool. The digestion reaction produces two different bands profiles when visualized on a 3% agarose gel according to the presence of the mutation at the heterozygous or homozygous state (Figure 3A). The results of the AluI digestion performed on our PCR product along with a negative control are shown in Figure 3B. Our sample digestion profile demonstrated the mutation to be in the heterozygous state, as the enzyme was able to recognize three cleavage sites on the wild-type sequence and two on the mutated one (51, 82, 171, 192, and 243 bp). A new, internal primer pair was able to confirm the NGS call (Figure 1J).

## Discussion

In this study, we showed that high-quality NGS data robustness could not benefit from Sanger validation due to errors occurring in the technology.

The two last decades witnessed the rapid and impressive advancements of high-throughput sequencing techniques, in terms of both experimental workflows and data-processing pipelines dedicated to the management of the large volumes of data generated by the various platforms. These technologies provided indeed unprecedented opportunities for the study and characterization of variants at the DNA and RNA level. Genetic information is, in fact, investigated at such a level of precision that the supporting role of these technologies for the study of complex hereditary phenotypes has been implemented in both research and diagnostics, also for the capability of different platforms to analyze and interrogate large gene panels, exomes, and genomes in times and costs that are progressively decreasing. Despite the attempt made by the European Guidelines (2016) (Matthijs et al., 2016) to provide the most useful indications to laboratories for the evaluation and validation of variants identified by NGS, these technologies still remain strictly dependent on computational tools and bioinformaticians for the highly complex data analysis, whose quality parameters may vary between laboratories as well as the pipelines used for alignment, variant calling, filtering, and annotation of variants. Current NGS guidelines do not define quality parameters or concrete guidance for confirmatory analysis. Therefore, Sanger sequencing is still considered the *gold standard* for the validation of NGS genetic variants and an essential step in the diagnostic routine. This kind of approach, however, raised a question about the actual cost-effectiveness of using very powerful platforms generating increasing quality data at progressively decreasing costs and the need to apply a “time-consuming” and not error-free [i.e., Allelic dropouts (ADOs)] technique, whose cost does not decrease as quickly over time, to validate the results.

Allelic dropout represents a significant cause of genotyping errors, potentially determining substantial negative consequences as it might lead to misdiagnosis of genetic diseases and false-negative/positive results depending on the allele that drops out (mutant or wild-type). ADO during PCR can be caused by a variety of mechanisms, and a number of factors influencing its rate have been described. Most ADO mechanisms are determined by the presence of a single-nucleotide variant (SNV) situated inside the primer-binding sequences on the targeted DNA, the SNV causing the failure of the primer-template annealing and the consequent amplification error. Concomitant presence of a differential allelic methylation and G-quadruplex motifs in some regions of the genome, DNA degradation leading to PCR refractory breaks, imperfect PCR conditions preventing DNA template accessibility, and presence of both homopolymer tracts and pseudogenes were also described as potential determinants of ADO or preferential amplification (Piyamongkol et al., 2003; Stevens et al., 2017).

In the current study, 942 out of 945 (99.7%) high-quality NGS variants identified in 218 subjects were validated by Sanger sequencing. Our data are in keeping with previous studies, suggesting that Sanger sequencing may not represent a necessary step to validate NGS variants when dealing with data meeting high-quality scores and an adequate depth of coverage. In fact, several previous studies evaluating data from different NGS platforms and approaches (targeted, exome, or whole-genome sequencing) identified almost 100% Sanger validation rate on a total of 14,495 variants (McCourt et al., 2013; Sikkema-Raddatz et al., 2013; Strom et al., 2014; Baudhuin et al., 2015; Beck et al., 2016; Zheng et al., 2019). In these studies, variants not validated by Sanger sequencing did not match adequate quality scores (Strom et al., 2014; Beck et al., 2016; Zheng et al., 2019). Both ours and literature data move in the direction of a limited usefulness of Sanger validation for NGS-derived variants associated with robust quality scores, suggesting a re-consideration of its application in routine diagnostics that should be limited to validation of a specific clinical phenotype-associated variant, quality assurance, and risk-avoidance purposes.

Beyond the previous issue, whether an accurate NGS approach is used, our data demonstrated that potential well-known failures affecting Sanger technology could further reduce its utility and instead determine higher costs and delay in analysis conclusion and laboratory report. In fact, among 945 variants, we identified three discrepant variants. Despite their high-quality parameters, namely, a balanced read number and high depths of coverage, Sanger validation failed in confirming NGS datum: in all three cases, NGS attributed a heterozygous state and Sanger sequencing, a mutated homozygous state in P1 and P3, and a wild-type homozygous state in P2. In the first case (*LTBP2* gene variant), the discrepancy was due to the presence of a small deletion in the DNA region binding the forward primer of the original pair. This deletion, potentially preventing the correct primer annealing during the initial amplification phase, had a frequency below 1% in the European ExAC population. This is of note as this rarity could presumably allow some of the online-automated primer-designing programs (which refer to dbSNP databases to omit common variants) as well as a manual approach to miss this kind of information during the primer construction and its inclusion in their sequence. This aspect is similar for private mutation of the patient in analysis. In fact, these variants evade the “masking” phase of the primer construction process where common variants are averted.

As concerns the further discrepant variant at the *LTBP2* locus, we speculated that this discrepancy might be the result of an alternative ADO phenomenon. In particular, the presence of SNV outside primer sequences (non-primer-binding-site SNVs) was demonstrated to promote a hairpin formation of the PCR products, this secondary structure preventing further amplification and extension failures (Lam and Mak, 2013). Lam and coworkers were in fact able to demonstrate that a heterozygous NGS deletion (*FAH*, NM_000137.1:c.1035_1037del) resulted in homozygosity at a first Sanger sequencing run due to a non-primer-binding-site SNV (*FAH*:c.961-35C, rs2043691) forming a strong hairpin structure and leading to amplification failure of the wild-type allele. Similarly, in our case, a non-primer-binding site SNP located outside the 3′ end of the original F primer (Figure 1H) was identified, which can be presumably held accountable due to the discrepancy we observed.

Regarding the third case, we were instead able to demonstrate that ADO had not occurred during the first amplification cycle via traditional PCR but exclusively within the direct sequencing reactions. Actually, even though Sanger sequencing by using the original primers pair showed a mutated homozygous state, RFLP procedure with the same primers showed the presence of both alleles of *TGFB1* mutation. The heterozygous state was confirmed at a second Sanger validation run with new primers. Our data do not define the fine mechanisms behind the ADO we observed in case number 3 during Sanger sequencing reaction; nevertheless, the previously mentioned mechanisms (apart from those involving SNV inside or proximal to the primer binding sequences) could be a potential explanation for the discrepancies we observed. These phenomena could also be impacted by intrinsic characteristics of Sanger technology which uses chain-terminating di-deoxynucleotides under suboptimal PCR conditions. Direct sequencing in fact employs a multiplex PCR ensuring the amplification of several distinct DNA fragments in a single reaction. This strategy necessarily operates under less stringent PCR conditions which might adversely affect amplification of individual loci or lead to secondary structure formation, thus invalidating the synthesis of the newly generated DNA strands (Piyamongkol et al., 2003).

## Conclusion

In conclusion, our data suggest the chance of Sanger approach errors that go beyond the presence of common variants. Actually, both studies of Beck et al. (2016) and Zheng et al. (2019) had also encountered this kind of criticism: in the first case, authors had to re-sequence 19 discrepant variants, 17 of which were validated after a second sequencing run as the first orthogonal Sanger validations were themselves incorrect. 17 of the NGS variants would have been considered false positives if a single round of Sanger sequencing were used as validation criteria. Zheng et al. (2019) were instead able to validate 98 high-quality NGS variants by mass spectrometry but not by Sanger sequencing, being some of those variants characterized by the presence of a homopolymer at the 100-bp flanking sequence or within pseudogenes. The authors suggested that if such practice were used in a clinical setting, more positive NGS variants would be discarded or incorrectly designated, when compared to using the NGS data directly. In fact, PCR-based amplification remains susceptible to ADO due to different mechanisms, such as private variants within primer-binding sites or secondary structure formation, potentially determining false-positive/negative results, heavily impacting genetic diagnosis of several diseases in the clinical setting. In addition to that, non-primer-binding-site SNVs have been demonstrated to have the ability to interfere with the PCR as well, making the primer designing process more laborious and time-consuming. Some genomic regions are also extremely difficult to amplify and might not yield high-quality Sanger results even after multiple attempts, thus possibly rendering the Sanger validation of a high-quality NGS variant not an adequate support. NGS still remains susceptible to errors, but, in our experience and according to those of many laboratories around the world, variants above the technology-dependent quality threshold are confirmed by Sanger in almost 100% of cases. Our study demonstrated that in case of discrepancy between a high-quality NGS variant and the subsequent Sanger validation, NGS call should not be *a priori* assumed to represent the source of the error. On the other hand, NGS approaches [targeted/whole-exome (WES)/genome sequencing (WGS)] exhibit some limitations, as they are characterized by a lack of uniformity in sequencing depth. In addition, due to capture of probe design matching the reference sequencing, preferentially enrichment of reference allele might represent a further NGS bias. Moreover, a further limitation of the NGS approach might be represented by the possible presence of false negatives (although reduced with the progressive improvement in experimental/computational analysis procedures) and in turn lack of information concerning genetic variants clinically relevant, even though standardized criteria are usually adopted (Sims et al., 2014; LaDuca et al., 2017; Dunn et al., 2018). Moreover, certain types of variants (copy number variations, large genomic rearrangements) remain difficult to detect by NGS, but experimental workflows, bioinformatic tools, and open-source software are being developed and constantly updated in order to overcome that issue (Shen and Seshan, 2016; Anwar et al., 2020; Han et al., 2020). Our paper is not designed to identify quality thresholds, due to limited number of genes investigated as compared to WES/WGS; nevertheless, further work of the scientific community on this task is needed. Hence, the necessity rises to determine a standardized, high-quality threshold that could be also established by reviewed International Guidelines for clinical genetic testing using NGS, thus possibly limiting Sanger confirmation to risk avoidance purposes (borderline conditions of variant quality parameters). This approach must be also weighed against increased cost and time of the genetic test and the potential failure to confirm a very high-quality NGS variant because of difficulties (ADO, unpredictable presence of private variants on primer-binding regions) of direct sequencing whose designation as “*gold standard*” should be reconsidered especially in light of the constantly implemented and accurate high-throughput technologies.

## Data Availability Statement

The datasets generated for this study can be found in NCBI GenBank IDs are: #2395939 (genomic DNA sequences), SUB8456066 (PRJNA674321 for high throughput sequencing reads. BioSample accessions: SAMN16634992, SAMN16634993, and SAMN16634994).

## Ethics Statement

The studies involving human participants were reviewed and approved by Ethics Committee of Careggi Hospital, Florence, Italy. The patients/participants provided their written informed consent to participate in this study.

## Author Contributions

RDC, ES, and BG proposed the concept of the study. RDC, ES, AK, and AV performed the patients’ genetic analysis and validations. AM and SS performed the bioinformatic analysis. RDC, BG, and ES prepared the manuscript. All authors critically reviewed the manuscript.

## Conflict of Interest

The authors declare that the research was conducted in the absence of any commercial or financial relationships that could be construed as a potential conflict of interest.

## Footnotes

1 https://sift.bii.a-star.edu.sg/

2 http://provean.jcvi.org/index.php

3 http://genetics.bwh.harvard.edu/pph2/

4 http://www.mutationtaster.org/

5 http://fathmm.biocompute.org.uk/

6 http://umd.be/Redirect.html

7 http://www.cbs.dtu.dk/services/NetGene2/

8 http://frodo.wi.mit.edu/primer3

9 https://genetools.org/SNPCheck/snpcheck.htm

10 https://www.ncbi.nlm.nih.gov/tools/primer-blast/

11 http://nc2.neb.com/NEBcutter2/
