# Supplementary material for: Sanger Validation of High-Throughput Sequencing in Genetic Diagnosis: Still the Best Practice?
Source: Front Genet. 2020 Dec 2;11:592588. doi: 10.3389/fgene.2020.592588 (PMC7738558; doi:10.3389/fgene.2020.592588)
Supplement: Supplementary file 1 [file Table_1.DOCX]

Supplementary Material


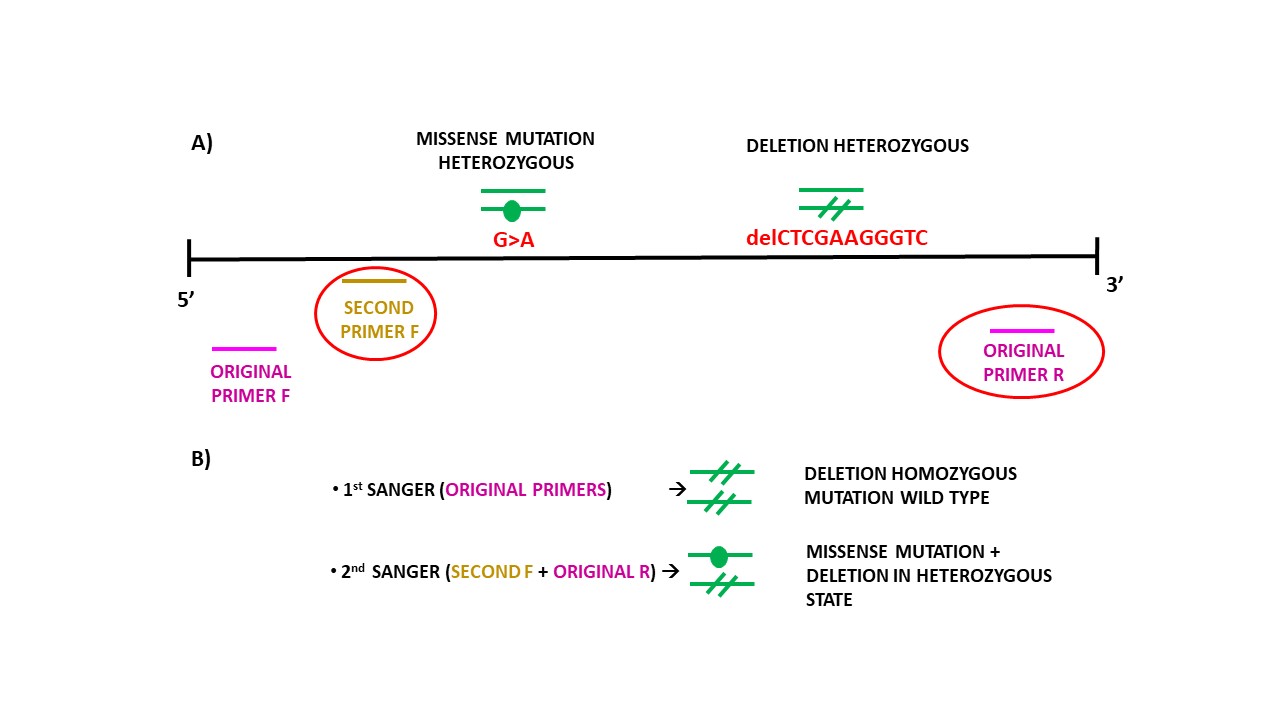


**Supplementary Figure 1.** Schematic representation of the point mutation and deletion variants on *LTBP2* gene (P2). The full green circle represents the point mutation, the two diagonal green bars represent the deletion (variants in-trans). Approximate localization of the original (purple) and second F (yellow) primers on the DNA sequence (horizontal black bar) is represented in panel (A). The 2 different primers combinations and the results of the relative electropherograms after direct sequencing are depicted in panel (B).

**Supplementary table S1.** Marfan syndrome and related disorders 97 genes panel

| Gene | Name | Cytogenetic location | OMIM |
| --- | --- | --- | --- |
| *COL11A1* | COLLAGEN, TYPE XI, ALPHA-1 | 1p21.1 | * 120280 |
| *MTHFR* | 5,10-METHYLENETETRAHYDROFOLATE REDUCTASE | 1p36.22 | * 607093 |
| *PLOD1* | PROCOLLAGEN-LYSINE, 2-OXOGLUTARATE 5-DIOXYGENASE | 1p36.22 | * 153454 |
| *B3GALT6* | BETA-1,3-GALACTOSYLTRANSFERASE 6 | 1p36.33 | * 615291 |
| *ADAMTSL4* | ADAMTS-LIKE 4 | 1q21.2 | * 610113 |
| *MFAP2* | MICROFIBRILLAR-ASSOCIATED PROTEIN 2 | 1p36.13 | * 156790 |
| *PTGS2* | PROSTAGLANDIN-ENDOPEROXIDE SYNTHASE 2 | 1q31.1 | * 600262 |
| *SKI* | V-SKI AVIAN SARCOMA VIRAL ONCOGENE HOMOLOG | 1p36.33-p36.32 | * 164780 |
| *TGFB2* | TRANSFORMING GROWTH FACTOR, BETA-2 | 1q41 | * 190220 |
| *CAPN2* | CALPAIN 2 | 1q41 | * 114230 |
| *AGT* | ANGIOTENSINOGEN | 1q42.2 | + 106150 |
| *MTR* | 5-METHYLTETRAHYDROFOLATE-HOMOCYSTEINE S-METHYLTRANSFERASE | 1q43 | * 156570 |
| *TGFBR3* | TRANSFORMING GROWTH FACTOR-BETA RECEPTOR, TYPE III; | 1p33-p32 | * 600742 |
| *TNXB* | TENASCIN XB | 6p21.33-p21.32 | * 600985 |
| *COL11A2* | COLLAGEN, TYPE XI, ALPHA-2 | 6p21.32 | * 120290 |
| *COL9A1* | COLLAGEN, TYPE IX, ALPHA-1 | 6q13 | * 120210 |
| *DSE* | DERMATAN SULFATE EPIMERASE | 6q22.1 | * 605942 |
| *FKBP14* | FK506-BINDING PROTEIN 14 | 7p14.3 | * 614505 |
| *FGF8* | FIBROBLAST GROWTH FACTOR 8 | 10q24.32 | * 600483 |
| *RET* | REARRANGED DURING TRANSFECTION PROTOONCOGENE | 10q11.21 | + 164761 |
| *ACTA2* | ACTIN, ALPHA-2, SMOOTH MUSCLE, AORTA | 10q23.31 | * 102620 |
| *B3GAT3* | BETA-1,3-GLUCURONYLTRANSFERASE 3 | 11q12.3 | * 606374 |
| *LTBP3* | LATENT TRANSFORMING GROWTH FACTOR-BETA-BINDING PROTEIN 3 | 11q13.1 | * 602090 |
| *EFEMP2/fbln4* | EGF-CONTAINING FIBULIN-LIKE EXTRACELLULAR MATRIX PROTEIN 2 | 11q13.1 | * 604633 |
| *LRP5* | LOW DENSITY LIPOPROTEIN RECEPTOR-RELATED PROTEIN 5 | 11q13.2 | * 603506 |
| *CCND1* | CYCLIN D1 | 11q13.3 | * 168461 |
| *LRP6* | LOW DENSITY LIPOPROTEIN RECEPTOR-RELATED PROTEIN 6 | 12p13.2 | * 603507 |
| *COL2A1* | COLLAGEN, TYPE II, ALPHA-1 | 12q13.11 | + 120140 |
| *LRP1* | LOW DENSITY LIPOPROTEIN RECEPTOR-RELATED PROTEIN 1 | 12q13.3 | * 107770 |
| *DCN* | DECORIN | 12q21.33 | * 125255 |
| *LTBP2* | LATENT TRANSFORMING GROWTH FACTOR-BETA-BINDING PROTEIN 2 | 14q24.3 | * 602091 |
| *TGFB3* | TRANSFORMING GROWTH FACTOR, BETA-3 | 14q24.3 | * 190230 |
| *FBLN5* | FIBULIN 5 | 14q32.12 | * 604580 |
| *ADAMTS17* | A DISINTEGRIN-LIKE AND METALLOPROTEINASE WITH THROMBOSPONDIN TYPE 1 MOTIF | 15q26.3 | * 607511 |
| *CHST14* | CARBOHYDRATE SULFOTRANSFERASE 14 | 15q15.1 | * 608429 |
| *FBN1* | FIBRILLIN 1 | 15q21.1 | * 134797 |
| *SMAD3* | MOTHERS AGAINST DECAPENTAPLEGIC, DROSOPHILA, HOMOLOG OF, 3 | 15q22.33 | * 603109 |
| *MYH11* | MYOSIN, HEAVY CHAIN 11, SMOOTH MUSCLE | 16p13.11 | * 160745 |
| *ABCC6* | ATP-BINDING CASSETTE, SUBFAMILY C, MEMBER 6 | 16p13.11 | * 603234 |
| *MAPK3* | MITOGEN-ACTIVATED PROTEIN KINASE 3 | 16p11.2 | * 601795 |
| *PDIA2* | PROTEIN DISULFIDE ISOMERASE, FAMILY A, MEMBER 2 | 16p13.3 | * 608012 |
| *AXIN1* | AXIS INHIBITOR 1 | 16p13.3 | * 603816 |
| *MMP2* | MATRIX METALLOPROTEINASE 2 | 16q12.2 | * 120360 |
| *CRYBA1* | CRYSTALLIN, BETA-A1 | 17q11.2 | * 123610 |
| *COL1A1* | CRYSTALLIN, BETA-A1 | 17q21.33 | * 123610 |
| *ACE* | ANGIOTENSIN I-CONVERTING ENZYME | 17q23.3 | + 106180 |
| *KCNJ2* | POTASSIUM CHANNEL, INWARDLY RECTIFYING, SUBFAMILY J, MEMBER 2 | 17q24.3 | * 600681 |
| *EMILIN2* | ELASTIN MICROFIBRIL INTERFACER 2 | 18p11.32 | * 608928 |
| *SMAD2* | MOTHERS AGAINST DECAPENTAPLEGIC, DROSOPHILA, HOMOLOG OF, 2 | 18q21.1 | * 601366 |
| *SMAD4* | MOTHERS AGAINST DECAPENTAPLEGIC, DROSOPHILA, HOMOLOG OF, 4 | 18q21.2 | * 600993 |
| *LTBP4* | LATENT TRANSFORMING GROWTH FACTOR-BETA-BINDING PROTEIN 4 | 19q13.2 | * 604710 |
| *TGFB1* | TRANSFORMING GROWTH FACTOR, BETA-1 | 19q13.2 | * 190180 |
| *ADAMTS10* | A DISINTEGRIN-LIKE AND METALLOPROTEINASE WITH THROMBOSPONDIN TYPE 1 MOTIF, 10 | 19p13.2 | * 608990 |
| *MMADHC* | MMADHC GENE | 2q23.2 | * 611935 |
| *ACVR1* | ACTIVIN A RECEPTOR, TYPE I | 2q24.1 | * 102576 |
| *COL3A1* | COLLAGEN, TYPE III, ALPHA-1 | 2q32.2 | * 120180 |
| *COL5A2* | COLLAGEN, TYPE V, ALPHA-2 | 2q32.2 | * 120190 |
| *FN1* | FIBRONECTIN 1 | 2q35 | * 135600 |
| *COL6A3* | COLLAGEN, TYPE VI, ALPHA-3 | 2q37.3 | * 120250 |
| *EMILIN1* | ELASTIN MICROFIBRIL INTERFACER 1 | 2p23.3 | * 130660 |
| *LTBP1* | LATENT TRANSFORMING GROWTH FACTOR-BETA-BINDING PROTEIN 1 | 2p22.3 | * 150390 |
| *JAG1* | JAGGED 1 | 20p12.2 | + 601920 |
| *EMILIN3* | ELASTIN MICROFIBRIL INTERFACER 3 | 20q12 | * 608929 |
| *MMP9* | MATRIX METALLOPROTEINASE 9 | 20q13.12 | * 120361 |
| *SLC2A10* | SOLUTE CARRIER FAMILY 2 (FACILITATED GLUCOSE TRANSPORTER), MEMBER 10 | 20q13.12 | * 606145 |
| *GATA5* | GATA-BINDING PROTEIN 5 | 20q13.33 | * 611496 |
| *CBS* | CYSTATHIONINE BETA-SYNTHASE | 21q22.3 | * 613381 |
| *COL6A1* | COLLAGEN, TYPE VI, ALPHA-1 | 21q22.3 | * 120220 |
| *COL6A2* | COLLAGEN, TYPE VI, ALPHA-2 | 21q22.3 | * 120240 |
| *UFD1L* | UBIQUITIN FUSION DEGRADATION 1-LIKE | 22q11.21 | * 601754 |
| *MAPK1* | MITOGEN-ACTIVATED PROTEIN KINASE 1 | 22q11.22 | * 176948 |
| *VHL* | VHL GENE | 3p25.3 | * 608537 |
| *ZPLD1* | ZONA PELLUCIDA-LIKE DOMAIN-CONTAINING PROTEIN 1 | 3q12.3 | * 615915 |
| *MYLK* | MYOSIN LIGHT CHAIN KINASE | 3q21.1 | * 600922 |
| *AGTR1* | ANGIOTENSIN RECEPTOR 1 | 3q24 | * 106165 |
| *PDCD10* | PROGRAMMED CELL DEATH 10 | 3q26.1 | * 609118 |
| *TGFBR2* | TRANSFORMING GROWTH FACTOR-BETA RECEPTOR, TYPE II | 3p24.1 | * 190182 |
| *FBN2* | FIBRILLIN 2 | 5q23.3 | * 612570 |
| *NKX2-5* | NK2 HOMEOBOX 5 | 5q35.1 | * 600584 |
| *B4GALT7* | BETA-1,4-GALACTOSYLTRANSFERASE 7 | 5q35.3 | * 604327 |
| *ADAMTS2* | A DISINTEGRIN-LIKE AND METALLOPROTEINASE WITH THROMBOSPONDIN TYPE 1 MOTIF, 2 | 5q35.3 | * 604539 |
| *AGGF1* | ANGIOGENIC FACTOR WITH G-PATCH AND FHA DOMAINS 1 | 5q13.3 | * 608464 |
| *MTRR* | METHIONINE SYNTHASE REDUCTASE | 5p15.31 | * 602568 |
| *NOS3* | NITRIC OXIDE SYNTHASE 3 | 7q36.1 | + 163729 |
| *HOXA1* | HOMEOBOX A1 | 7p15.2 | * 142955 |
| *CCM2* | CCM2 GENE | 7p13 | * 607929 |
| *KRIT1* | KREV INTERACTION TRAPPED | 7q21.2 | * 604214 |
| *COL1A2* | COLLAGEN, TYPE I, ALPHA-2 | 7q21.3 | * 120160 |
| *TGFBR1* | TRANSFORMING GROWTH FACTOR-BETA RECEPTOR, TYPE I | 9q22.33 | * 190181 |
| *PTGS1* | PROSTAGLANDIN-ENDOPEROXIDE SYNTHASE 1 | 9q33.2 | * 176805 |
| *ENG* | ENDOGLIN | 9q34.11 | * 131195 |
| *COL5A1* | COLLAGEN, TYPE V, ALPHA-1 | 9q34.3 | * 120215 |
| *NOTCH1* | NOTCH, DROSOPHILA, HOMOLOG OF, 1 | 9q34.3 | * 190198 |
| *GNAQ* | GUANINE NUCLEOTIDE-BINDING PROTEIN, Q POLYPEPTIDE | 9q21.2 | * 600998 |
| *AGTR2* | ANGIOTENSIN II RECEPTOR, TYPE 2; | Xq23 | * 300034 |
| *FLNA* | FILAMIN A | Xq28 | * 300017 |
| *ELN* | ELASTIN | 7q11.23 | * 130160 |

**Supplementary Table S2.** Familial dyslipidemia 57 genes panel

| Gene | Name | Cytogenetic location | OMIM |
| --- | --- | --- | --- |
| *LDLRAP1* | LOW DENSITY LIPOPROTEIN RECEPTOR ADAPTOR PROTEIN 1 | 1p36.11 | * 605747 |
| *PCSK9* | PROPROTEIN CONVERTASE, SUBTILISIN/KEXIN-TYPE, 9 | 1p32.3 | * 607786 |
| *ANGPTL3* | ANGIOPOIETIN-LIKE 3 | 1p31.3 | * 604774 |
| *CELSR2* | CADHERIN EGF LAG SEVEN-PASS G-TYPE RECEPTOR 2 | 1p13.3 | * 604265 |
| *APOA2* | APOLIPOPROTEIN A-II DEFICIENCY, INCLUDED | 1q23.3 | + 107670 |
| *APOB* | APOLIPOPROTEIN B | 2p24.1 | * 107730 |
| *GCKR* | GLUCOKINASE REGULATORY PROTEIN | 2p23.3 | * 600842 |
| *ABCG5* | ATP-BINDING CASSETTE, SUBFAMILY G, MEMBER 5 | 2p21 | * 605459 |
| *ABCG8* | ATP-BINDING CASSETTE, SUBFAMILY G, MEMBER 8 | 2p21 | * 605460 |
| *INSIG2* | INSULIN-INDUCED GENE 2 | 2q14.1-q14.2 | * 608660 |
| *ITIH4* | INTER-ALPHA-TRYPSIN INHIBITOR, HEAVY CHAIN 4 | 3p21.1 | * 600564 |
| *STAP1* | SIGNAL TRANSDUCING ADAPTOR FAMILY MEMBER 1 | 4q13.2 | * 604298 |
| *ABCG2* | ATP-BINDING CASSETTE, SUBFAMILY G, MEMBER 2 | 4q22.1 | * 603756 |
| *MTTP* | MICROSOMAL TRIGLYCERIDE TRANSFER PROTEIN | 4q23 | * 157147 |
| *DAB2* | DAB ADAPTOR PROTEIN 2 | 5p13.1 | * 601236 |
| *GHR* | GROWTH HORMONE RECEPTOR | 5p13-p12 | * 600946 |
| *HMGCR* | 3-HYDROXY-3-METHYLGLUTARYL-CoA REDUCTASE | 5q13.3 | + 142910 |
| *SAR1B* | SECRETION-ASSOCIATED RAS-RELATED GTPase 1B | 5q31.1 | * 607690 |
| *MYLIP* | MYOSIN REGULATORY LIGHT CHAIN-INTERACTING PROTEIN | 6p22.3 | * 610082 |
| *HFE* | HOMEOSTATIC IRON REGULATOR | 6p22.2 | * 613609 |
| *BTN2A1* | BUTYROPHILIN, SUBFAMILY 2, MEMBER A1 | 6p22.2 | * 613590 |
| *SLC22A1* | SOLUTE CARRIER FAMILY 22 (ORGANIC CATION TRANSPORTER), MEMBER 1 | 6q25.3 | * 602607 |
| *LPA* | LIPOPROTEIN(a) | 6q25-q26 | * 152200 |
| *PPP1R17* | PROTEIN PHOSPHATASE 1 REGULATORY SUBUNIT 17 | 7p14.3 | * 604088 |
| *NPC1L1* | NPC1-LIKE 1 | 7p13 | * 608010 |
| *ABCB1* | ATP-BINDING CASSETTE, SUBFAMILY B, MEMBER 1 | 7q21.12 | * 171050 |
| *PON1* | PARAOXONASE 1 | 7q21.3 | + 168820 |
| *LPL* | LIPOPROTEIN LIPASE | 8p21.3 | * 609708 |
| *EPHX2* | EPOXIDE HYDROLASE 2, CYTOSOLIC | 8p21.2-p21.1 | * 132811 |
| *GPIHBP1* | GLYCOSYLPHOSPHATIDYLINOSITOL-ANCHORED HIGH DENSITY LIPOPROTEIN-BINDING PROTEIN 1 | 8q24.3 | * 612757 |
| *DGAT1* | DIACYLGLYCEROL O-ACYLTRANSFERASE 1 | 8q24.3 | * 604900 |
| *ABCA1* | ATP-BINDING CASSETTE, SUBFAMILY A, MEMBER 1 | 9q31.1 | * 600046 |
| *CH25H* | CHOLESTEROL 25-HYDROXYLASE | 10q23.31 | * 604551 |
| *OSBPL5* | OXYSTEROL-BINDING PROTEIN-LIKE PROTEIN 5 | 11p15.4 | * 606733 |
| *APOA5* | APOLIPOPROTEIN A-V | 11q23.3 | * 606368 |
| *APOA4* | APOLIPOPROTEIN A-IV | 11q23.3 | * 107690 |
| *APOC3* | APOLIPOPROTEIN C-III | 11q23.3 | * 107720 |
| *APOA1* | APOLIPOPROTEIN A-I | 11q23.3 | * 107680 |
| *ST3GAL4* | ST3 BETA-GALACTOSIDE ALPHA-2,3-SIALYLTRANSFERASE 4 | 11q24.2 | * 104240 |
| *SLCO1B1* | SOLUTE CARRIER ORGANIC ANION TRANSPORTER FAMILY, MEMBER 1B1 | 12p12.1 | * 604843 |
| *GPD1* | GLYCEROL-3-PHOSPHATE DEHYDROGENASE 1 | 12q13.12 | * 138420 |
| *LRP1* | LOW DENSITY LIPOPROTEIN RECEPTOR-RELATED PROTEIN 1 | 12q13.3 | * 107770 |
| *SCARB1* | SCAVENGER RECEPTOR CLASS B, MEMBER 1 | 12q24.31 | * 601040 |
| *NYNRIN* | NYN Domain And Retroviral Integrase Containing | 14q12 |  |
| *NPC2* | EPIDIDYMAL SECRETORY PROTEIN | 14q24.3 | * 601015 |
| *LIPC* | LIPASE, HEPATIC | 15q21.3 | * 151670 |
| *LMF1* | LIPASE MATURATION FACTOR 1 | 16p13.3 | * 611761 |
| *CETP* | CHOLESTERYL ESTER TRANSFER PROTEIN, PLASMA | 16q13 | * 118470 |
| *LCAT* | LECITHIN:CHOLESTEROL ACYLTRANSFERASE | 16q22.1 | * 606967 |
| *SREBF1* | STEROL REGULATORY ELEMENT-BINDING TRANSCRIPTION FACTOR 1 | 17p11.2 | * 184756 |
| *NPC1* | NPC1 GENE | 18q11.2 | * 607623 |
| *CREB3L3* | cAMP RESPONSE ELEMENT-BINDING PROTEIN 3-LIKE 3 | 19p13.3 | * 611998 |
| *LDLR* | LOW DENSITY LIPOPROTEIN RECEPTOR | 19p13.2 | * 606945 |
| *APOE* | APOLIPOPROTEIN E | 19q13.32 | * 107741 |
| *APOC2* | APOLIPOPROTEIN C-II | 19q13.32 | * 608083 |
| *LIPI* | LIPASE I | 21q11.2 | * 609252 |
| *SREBF2* | STEROL REGULATORY ELEMENT-BINDING TRANSCRIPTION FACTOR 2 | 22q13.2 | * 600481 |

**Supplementary Table S3.** von Willebrand Disease 10 genes panel

| Gene | Name | Cytogenetic location | OMIM |
| --- | --- | --- | --- |
| *ITGB3* | INTEGRIN, BETA-3 | 17q21.31 | * 173470 |
| *VWF* | VON WILLEBRAND FACTOR | 12p13.31 | * 613160 |
| *ADAMTS13* | A DISINTEGRIN-LIKE AND METALLOPROTEASE WITH THROMBOSPONDIN TYPE 1 MOTIF, 13 | 9q34.2 | * 604134 |
| *F8* | COAGULATION FACTOR VIII | Xq28 | * 300841 |
| *GP1BA* | GLYCOPROTEIN Ib, PLATELET, ALPHA POLYPEPTIDE | 17p13.2 | * 606672 |
| *GP1BB* | GLYCOPROTEIN Ib, PLATELET, BETA POLYPEPTIDE | 22q11.21 | * 138720 |
| *GP5* | GLYCOPROTEIN V, PLATELET | 3q29 | * 173511 |
| *GP9* | GLYCOPROTEIN IX, PLATELET | 3q21.3 | * 173515 |
| *ITGA2B* | INTEGRIN, ALPHA-2B | 17q21.31 | * 607759 |
| *P2RY12* | PURINERGIC RECEPTOR P2Y, G PROTEIN-COUPLED, 12 | 3q25.1 | * 600515 |

**Supplementary Table S4:** PCR primer sequences

|  | Primer sequence |
| --- | --- |
| P1 | LTBP2_27F: 5’- agcagagaacgagagagtgt -3’  LTBP2_27R: 5’- gggcgaggagttgaagtct -3’  LTBP2_27F_New: 5’- GGGTTGAGAGGCTGCTTTTC -3’  LTBP2_27R_New: 5’- CAGGACCAGTTGAGGAGGAG -3’ |
| P2 | LTBP2_29F: 5’- TCCCTGCTGCACTGCTTAAT -3’  LTBP2_29R: 5’- TCAGCTATGTAGAGAGGCGT -3’  LTBP2_29F_SECOND: 5’- cttcttagggttgtggagagc -3’  LTBP2_INTRON28F: 5’- GGAAACCCTGATGCTGACTT -3’  LTBP2_INTRON28R: 5’- ACAACCCTAAGAAGTGGCCT -3’ |
| P3 | TGFB1_1F: 5’- accacaccagccctgttc -3’  TGFB1_1R: 5’- tctgccagtcacttcctacc -3’  TGFB1_1F_INTERNAL: 5’- GCAAGACTATCGACATGGAGC -3’  TGFB1_1R_INTERNAL: 5’- CGTAGTAGTCGGCCTCAGG -3’ |
